# Association between Diabetic Retinopathy and Chronic Periodontitis—A Cross-Sectional Study

**DOI:** 10.3390/medsci6040104

**Published:** 2018-11-23

**Authors:** Veena H. R., Sribhargava Natesh, Sudhir R. Patil

**Affiliations:** 1 Department of Periodontology, K.L.E Society’s Institute of Dental Sciences, Bangalore 560 022, Karnataka, India; zonaperio@gmail.com; 2 Vitreoretina, Neuro-Ophthalmology and Ocular Oncology, Nethra Eye Hospital, Bangalore 560 094, India; sribhargava.natesh@gmail.com; 3 Drishti Eye Hospitals, Raichur 562 110, Karnataka, India

**Keywords:** periodontal disease, diabetes mellitus, diabetic retinopathy

## Abstract

Periodontal disease (PD), a chronic inflammatory condition characterized by destruction of the supporting tissues of the teeth, increases the risk of complications in diabetics. Diabetic retinopathy (DR) is a microvascular complication of prolonged hyperglycaemia. There appears to be a similarity in the pathogenesis of DR and PD. Hence, this study aimed to investigate the association, if any, between DR and PD, correlate the severity of DR with the severity of PD, and investigate the association between glycated haemoglobin (HbA1c), serum creatinine and periodontal variables. The periodontal status of 200 adult diabetic patients in the age group of 30–65 years with varying severity of DR was assessed. Evaluation of the severity of PD was assessed by recording clinical parameters. Haematological investigations including glycated haemoglobin (HbA1c) and serum creatinine were estimated before the initiation of treatment for DR. A statistically significant association between the mean duration of diabetes mellitus (DM) and the severity of DR and PD was found. The severity of PD was directly correlated with the severity of DR. There was a significant association between the levels of HbA1c and serum creatinine and severity of DR and PD. There could be a plausible relationship between DR and PD. Further prospective studies on a larger population with longer follow-ups are required to ascertain whether PD and its severity directly affect the progression and severity of DR.

## 1. Introduction

Diabetes mellitus (DM) is a metabolic disorder marked by high levels of blood glucose resulting from a defect in insulin production, insulin action or both. The complications of prolonged hyperglycaemia can be classified into macrovascular (coronary artery disease, peripheral arterial disease, and stroke) and microvascular (nephropathy, neuropathy, and retinopathy) [1].

According to the world health organization (WHO), the prevalence of DM in adults worldwide was estimated to be 4% in 1995 and is predicted to rise to 5.4% by 2025 [2]. According to the recently published ICMR–INDIAB diabetes prevalence study, the overall prevalence of DM in India is estimated to be 7.3% and the prevalence of pre-diabetes 10.3% based on the WHO criteria or 24.7% based on the American dental association ADA criteria [3]. Diabetes mellitus is also beginning to appear much earlier in life, and chronic long-term complications are becoming more common [4].

Diabetic retinopathy (DR), a microvascular complication of chronic DM, is estimated to be the most frequent cause of new cases of blindness among adults aged 20–74 years [5]. It is estimated that almost half of the world’s population of diabetics will develop a degree of DR at some point in their lifetime. The incidence and risk of progression of DR have declined during the past 30 years, from 90% to less than 50%, due to improvements in medical care [6].

Diabetic retinopathy is broadly classified as either background or proliferative. Background retinopathy includes features such as small haemorrhages in the middle layers of the retina. They appear clinically as red dots during retinal examination. Retinal oedema may result from microvascular leakage and is indicative of a compromised blood‒retinal barrier. Retinal oedema may require intervention because it is sometimes associated with visual deterioration [7,8].

Proliferative retinopathy is characterized by the formation of new blood vessels on the surface of the retina. If proliferation continues, it can lead to blindness through vitreous haemorrhage and retinal detachment. Laser photocoagulation can often prevent proliferative retinopathy from progressing to blindness. Hence, close surveillance for the existence or progression of retinopathy in patients with diabetes is crucial [8].

Periodontal disease (PD) is a chronic inflammatory condition characterized by destruction of the periodontal tissues, resulting in loss of connective tissue attachment, loss of alveolar bone, and the formation of pathological pockets around the diseased teeth. Several studies have explored the association between PD and DM, with inflammation acting as a common pathologic entity for both diseases [9]. It is generally accepted that PD is highly prevalent and more severe in subjects with Type II DM than in non-diabetics [1]. The chronic bacterial challenge in periodontal disease is a constant source of inflammatory mediators that may be associated with insulin resistance, which in turn increases the risk of complications of DM [10]. Periodontal signs and symptoms are now recognized as the sixth complication of DM [11]. 

The association of PD and the development of DR can be explained by various mechanisms. Elevated levels of interleukin 6 (IL-6), C-reactive protein (CRP) and fibrinogen in PD increase insulin resistance [12]. Periodontal disease-induced oxidative stress results in tissue damage and cell death [13]. It has been reported that PD leads to a progressive increase in levels of vascular endothelial growth factor (VEGF) in the gingival crevicular fluid [14]. Elevated levels of VEGF secondary to PD can affect the blood-retinal barrier, leading to retinal pathology [15]. Periodontal disease can also lead to atherosclerosis, which may result in hypoxia in the retina, causing proliferation of new fragile and leaky blood vessels, leading to retinal destruction and eventually retinal detachment [8,16].

On the basis of the pathological similarity between diabetic microvascular changes that may occur in the retina and periodontal tissues, the present study was conducted with the following aims and objectives.

1. To investigate the association between PD and DR.
2. To investigate the correlation, if any, between the severity of DR and the severity of PD.
3. To investigate the association between glycated haemoglobin (HbA1c), serum creatinine and periodontal variables.

## 2. Methodology

### 2.1. Data Source

The clinical material for this prospective analytical cross-sectional study was accessed by the Retina facility at Nethra eye hospital, Bangalore, Karnataka, India. Included in the study were 259 adult diabetic patients (160 males and 99 females) in the age group of 30–65 years, medically diagnosed with Type II DM. All subjects gave written consent after receiving information concerning the research objectives. The study was approved by the institute ethics committee (Approval no. KIDS/IEC/03-2015/6).

**Figure 1:**
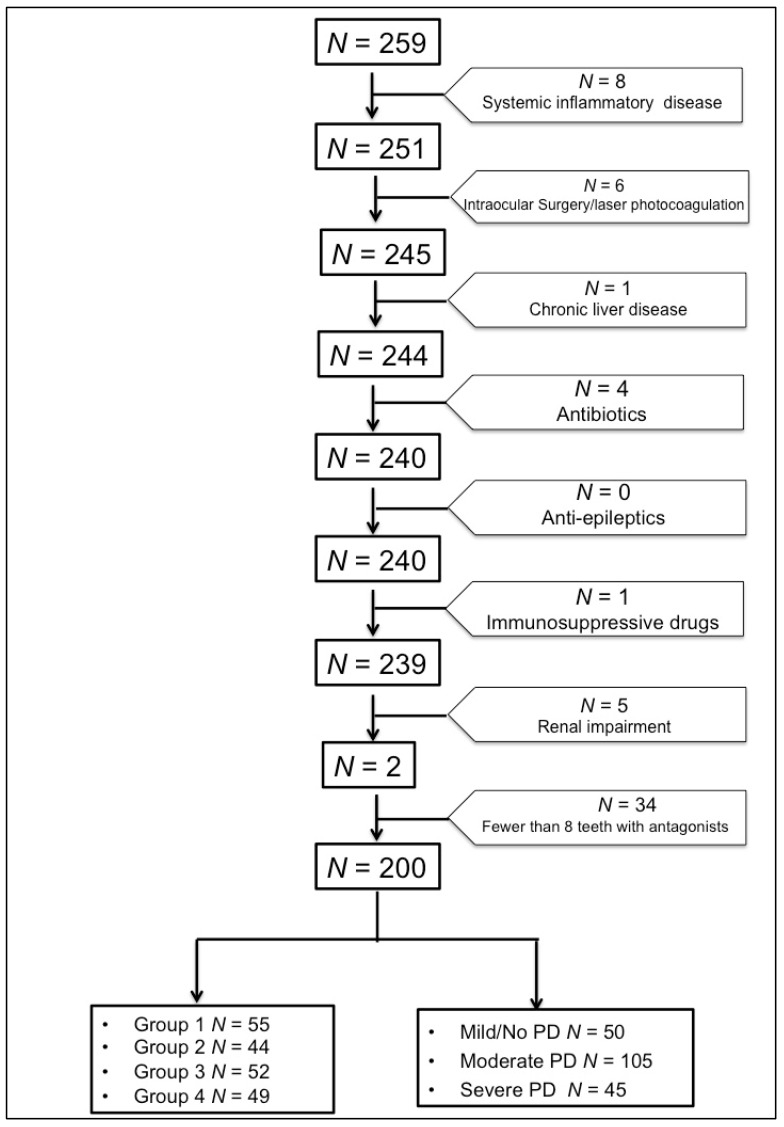
Flowchart describing the derivation of study samples for statistical analyses. PD—Periodontal disease.

Subjects with fewer than eight teeth with antagonists, the presence of any systemic inflammatory diseases, a history of intraocular surgery or previous laser photocoagulation, renal impairment (serum creatinine: 130 mol/L), chronic liver disease, or receiving medical treatment that could influence the studied parameters such as antibiotics, antiepileptic, or immunosuppressive drugs at the time of the study were excluded. Based on the above exclusion criteria, out of the 259 subjects screened, 59 subjects were excluded and the data from the remaining 200 subjects (144 males and 56 females) were considered for statistical analysis (Figure 1). 

The study subjects were screened for DR by a trained ophthalmologist. Based on the severity of DR, the subjects were divided into four groups [17]:

**Group 1:** Mild to moderate non-proliferative diabetic retinopathy
**Group 2:** Moderate to severe non-proliferative diabetic retinopathy
**Group 3:** Proliferative diabetic retinopathy
**Group 4:** Control group with normal fundus on examination.

The ocular examination was followed by clinical periodontal examination by a trained periodontist. The clinical parameters measured were plaque index (PI) (Loe and Silness) [18], gingival index (GI) (Loe and Silness) [18], probing pocket depth (PPD) and clinical attachment level (CAL). Probing pocket depth and clinical attachment level are clinical measures for quantifying periodontal attachment loss and were recorded to the nearest millimetre using a William’s graduated periodontal probe [19]. The severity of PD was classified as follows [19]:

(a) **Severe periodontitis**: ≥2 sites with interproximal CAL ≥ 6 mm (not on the same tooth) and ≥1 interproximal site with PPD ≥ 5 mm.

(b) **Moderate periodontitis**: ≥2 sites with interproximal CAL ≥ 4 mm (not on the same tooth) or ≥2 interproximal sites with PPD ≥ 5 mm (not on the same tooth).

(c) **Mild or no periodontitis**: neither severe nor moderate periodontitis.

Haematological investigations including glycated haemoglobin (HbA1c) and serum creatinine were done before the treatment for DR was initiated. 

### 2.2. Statistical Analysis 

One-way analysis of variance was used to test the difference between groups. If the ‘F’ value was significant it indicated a significant difference between the group means. To find out which of the two groups means was significantly different, the post hoc Tukey test was used. If the F value was not significant it indicated that there was no significant difference between the groups and the analysis was stopped at that stage and did not continue to the Tukey test. Student’s *t*-test was used to determine whether there was a statistically significant difference between the groups in the parameters measured. The proportion was measured using a Chi square test. A *p* value of less than 0.05 was accepted as indicating statistical significance. Data analysis was carried out using Statistical Package for Social Sciences (SPSS version 10.5). 

## 3. Results

**Figure 2:**
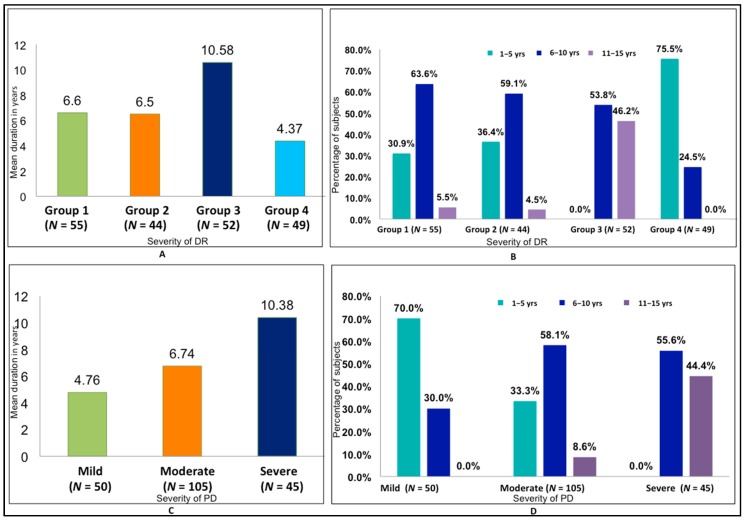
Association between mean duration of diabetic retinopathy (DR) and PD with severity of disease. (**A**) Comparison of mean duration of diabetes mellitus (DM) with severity of DR. (**B**) Distribution of duration of DM by severity of DR. (**C**) Comparison of mean duration of DM with severity of PD. (**D**) Distribution of duration of DM by severity of PD.

**Figure 3:**
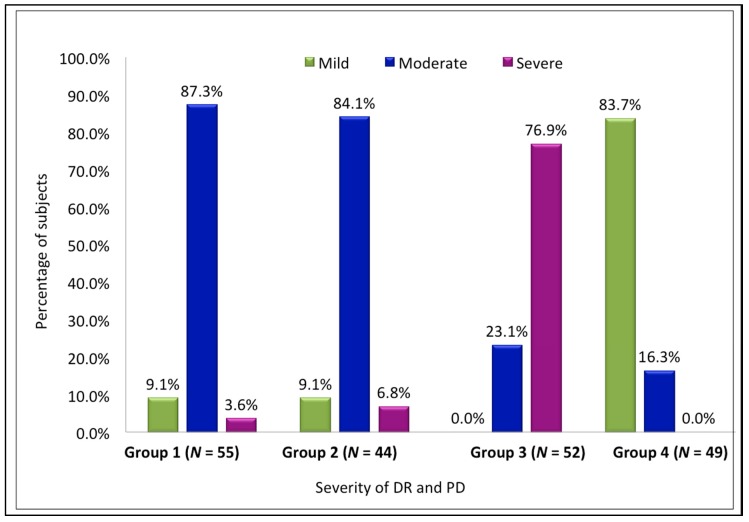
Association between severity of DR and severity of PD.

**Figure 4:**
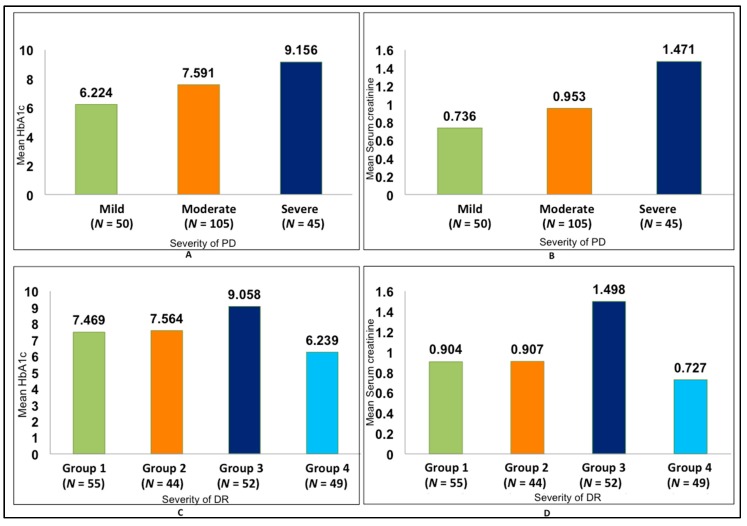
Association between glycated haemoglobin (HbA1c) and serum creatinine with DR and PD. (**A**) Comparison of mean HbA1c with PD. (**B**) Comparison of mean serum creatinine with PD. (**C**) Comparison of mean HbA1c with DR. (**D**) Comparison of mean serum creatinine with DR.

The relationship between the duration of DM (in years) and the severity of DR was found to be statistically significant (*p* < 0.001) (Figure 2). Both the intragroup and intergroup comparisons of the association between the mean PI and GI scores and severity of DR were also found to be statistically significant (*p* < 0.001), with the highest scores in Group 3 and the lowest in Group 4 (Table 1 and Table 2). The association between duration of DM and severity of PD was also found to be statistically significant (*p* < 0.001), with a mean duration of 10.3 years in severe periodontitis and 4.76 years in mild periodontitis (Figure 2). A statistically significant correlation between the severity of DR and severity of PD was also found (*p* < 0.001) (Figure 3, Table 3). There was a positive correlation between the severity of periodontal disease and levels of HbA1c and serum creatinine, with a statistically significant difference between the groups (*p* < 0.001) (Figure 4, Table 4). A statistically significant association (*p* < 0.001) was observed between the severity of diabetic retinopathy and levels of HbA1c and serum creatinine, with a mean HbA1c value of 9.058% and mean serum creatinine level of 1.498 mg/dL in Group 3 (Figure 4).

## 4. Discussion

The present prospective cross-sectional study involved 200 adult diabetic patients (144 males and 56 females), of whom 151 presented with DR of varying severity. In our study, the mean duration of type II DM was directly correlated with the severity of DR. A similar association was found in a study by Amiri et al., who reported a mean duration of DM of 10.5 years [6]. A similar correlation was also found in another study by Noma et al., in which the mean duration of DM reported was 14.3 years [20].

The mean PI and GI scores were highest in patients with proliferative DR. A positive correlation was found between the duration of DM and severity of PD. Banthia et al. demonstrated that subjects with DR had poorer oral hygiene compared to subjects with DM and no DR [21]. In the presence of periodontal disease, complications of DM can occur early and periodontal disease can place diabetics at an increased risk of developing ocular complications [22]. Type II diabetics with retinopathy are five times more likely to develop periodontal disease than type II diabetics without retinopathy. DM is associated with PD and the outcome of periodontal destruction is significantly more severe in subjects with DR [22]. Bajaj et al. reported that retinal microvascular complications of DM were found in 50% of cases with PD [23]. Another study in a Korean population reported a statistically significant increase in the prevalence of periodontitis in individuals who had proliferative DR [24].

The severity of PD strongly correlated with HbA1c and serum creatinine levels in the present study. Several studies have attributed poor glycaemic control to chronic periodontal infections, which in turn increases the risk of complications. Poorly controlled diabetics are at a greater risk for severe forms of periodontal disease [25,26]. A high prevalence of PD among individuals during early stages of chronic kidney disease and end-stage renal disease has also been reported [27]. A study conducted by Tonetti et al. showed improvement in endothelial function, with aggressive treatment of PD by a decrease in the inflammatory burden [28]. Several studies have stated that patients with severe periodontitis had statistically significant elevated serum CRP compared to those without periodontitis [29,30]. A study performed by Navarro et al. demonstrated the association between urinary albumin excretion and inflammatory factors in the patients with Type II DM [31]. Cintra et al. reported elevated serum creatinine levels in diabetic rats in the presence of periodontal and pulpal disease [32]. There are also reports of the duration of diabetes and high serum creatinine levels along with cholesterol, and triglycerides being significantly associated with the occurrence of proliferative retinopathy [33]. Chronic kidney disease defined by a triple marker panel (serum creatinine, serum cystatin C, and albuminuria) was strongly associated with moderate DR in an Asian population with diabetes [34].

### Limitations of the Study 

This was a cross-sectional study limited to 200 subjects to minimize confounding factors. Not all the confounding factors for PD were considered in the current study. Further prospective studies on a larger population with longer follow-ups are required to ascertain whether PD and its severity directly affect the progression and severity of DR.

## 5. Conclusions 

As this is a cross-sectional study with a limited sample size, a probable cause and effect relationship between PD and DR cannot be established. Whether PD is a risk factor for DR or a coincidental finding and whether the severity of PD influences the severity and progression of DR remains to be determined by prospective studies on a larger population and with longer follow-ups. However, prevention and control of PD must be considered an integral part of diabetes management strategies. Periodontal medicine promotes a strong collaboration between dental and medical professionals, which implies better communication and an effective team approach in clinical practice. Moreover, the dentist should be aware of the risk of DR while examining diabetic patients with PD and should consider referring such patients to an ophthalmologist for fundus examination. This might not only reduce the systemic burden but also improve the oral environment.

## Figures and Tables

**Table 1:** Comparison of Plaque index and Gingival index scores with severity of DR. SD—standard deviation.

Plaque Index (Silness and Loe)	N	Mean	SD	Min.	Max.	‘F’ Value	*p* Value
Group 1	55	1.345	0.5381	0.4	2.3	40.633	<0.001
Group 2	44	1.530	0.5398	0.4	2.6		
Group 3	52	2.287	0.4606	0.6	3.0		
Group 4	49	1.359	0.4721	0.5	2.0		
**Gingival index** **(Loe and Silness)**							
Group 1	54	1.504	0.4148	0.7	2.1	61.554	<0.001
Group 2	43	1.730	0.3668	0.7	2.6		
Group 3	52	2.381	0.2552	1.7	2.9		
Group 4	49	1.622	0.3853	0.8	2.6		

**Table 2:** Pairwise comparison between the groups for Plaque index and Gingival index. SE—standard error.

		Mean Difference	SE of Diff	*p* Value
**Plaque Index**	Group 1 vs. Group 2	−0.184	0.102	0.273
	Group 1 vs. Group 3	−0.941	0.097	<0.001
	Group 1 vs. Group 4	−0.014	0.099	0.999
	Group 2 vs. Group 3	−0.757	0.103	<0.001
	Group 2 vs. Group 4	0.170	0.105	0.365
	Group 3 vs. Group 4	0.927	0.100	*<0.001*
**Gingival Index**	Group 1 vs. Group 2	−0.227	0.074	0.013
	Group 1 vs. Group 3	−0.877	0.070	<0.001
	Group 1 vs. Group 4	−0.119	0.071	0.343
	Group 2 vs. Group 3	−0.651	0.074	<0.001
	Group 2 vs. Group 4	0.108	0.075	0.482
	Group 3 vs. Group 4	0.758	0.072	<0.001

**Table 3:** Association between severity of PD with severity of DR.

	Severity of Periodontal Disease			Total	Χ^2^	*p* Value
	Mild	Moderate	Severe			
**Group 1**	5	48	2	55	225.301	<0.001
	9.1%	87.3%	3.6%	100.0%		
**Group 2**	4	37	3	44		
	9.1%	84.1%	6.8%	100.0%		
**Group 3**	0	12	40	52		
	0.0%	23.1%	76.9%	100.0%		
**Group 4**	41	8	0	49		
	83.7%	16.3%	0.0%	100.0%		
**Total**	50	105	45	200		
	25.0%	52.5%	22.5%	100.0%		

**Table 4:** Pairwise comparison between groups for severity of PD, HbA1c and Serum creatinine.

		Mean Difference	SE of Diff	*p* Value
**Severity of PD**	**Mild vs. Moderate**	−1.983	0.392	<0.001
	**Mild vs. Severe**	−5.618	0.468	<0.001
	**Moderate vs. Severe**	−3.635	0.406	<0.001
**HbA1c**	**Mild vs. Moderate**	−1.367	0.160	<0.001
	**Mild vs. Severe**	−2.932	0.191	<0.001
	**Moderate vs. Severe**	−1.565	0.166	<0.001
**Serum Creatinine**	**Mild vs. Moderate**	−0.217	0.056	<0.001
	**Mild vs. Severe**	−0.735	0.067	<0.001
	**Moderate vs. Severe**	−0.518	0.058	<0.001
